# Functional proteomics-aided selection of protease inhibitors for herbivore insect control

**DOI:** 10.1038/srep38827

**Published:** 2016-12-13

**Authors:** Asieh Rasoolizadeh, Aurélie Munger, Marie-Claire Goulet, Frank Sainsbury, Conrad Cloutier, Dominique Michaud

**Affiliations:** 1 Département de phytologie, Université Laval, Québec City, QC, Canada; 2 Département de biologie, Université Laval, Québec City QC, Canada

## Abstract

Studies have reported the potential of protease inhibitors to engineer insect resistance in transgenic plants but the general usefulness of this approach in crop protection still remains to be established. Insects have evolved strategies to cope with dietary protease inhibitors, such as the use of proteases recalcitrant to inhibition, that often make the selection of effective inhibitors very challenging. Here, we used a functional proteomics approach for the ‘capture’ of Cys protease targets in crude protein extracts as a tool to identify promising cystatins for plant improvement. Two cystatins found to differ in their efficiency to capture Cys proteases of the coleopteran pest *Leptinotarsa decemlineata* also differed in their usefulness to produce transgenic potato lines resistant to this insect. Plants expressing the most potent cystatin at high level had a strong repressing effect on larval growth and leaf intake, while plants expressing the weakest cystatin showed no effect on both two parameters compared to untransformed parental line used for genetic transformation. Our data underline the relevance of considering the whole range of possible protease targets when selecting an inhibitor for plant pest control. They also confirm the feasibility of developing cystatin-expressing transgenics resistant to a major pest of potato.

Three papers have described, almost 30 years ago, the potential of plant genetic transformation to implement insect resistance into crop genomes. Two of those papers, by Vaeck *et al*.^1^ and Fischhoff *et al*.^2^, reported on the potential of Cry toxin-encoding genes from the soil bacterium *Bacillus thuringiensis* (Bt) to produce transgenic plant lines resistant to the tobacco hornworm *Manduca sexta*. The third paper, by Hilder *et al*.^3^, discussed the potential of a trypsin inhibitor from cowpea to produce transgenic lines resistant to another lepidopteran pest, the tobacco budworm *Heliothis virescens*. These three seminal papers were followed by hundreds of reports addressing various questions on insect-resistant transgenic crops, notably related to the large-scale deployment and durable use of ‘Bt crops’ worldwide^4^,^5^,^6^ or to the basic reasons for the mitigated efficiency and still limited use of protease inhibitors in plant protection^7^,^8^.

One explanation for the commercial success of Bt crops over protease inhibitor-expressing crops most likely lies in the different modes of action and pesticidal efficiency of the expressed proteins in agricultural contexts^9^. A second explanation is the natural ability of herbivore pests to elude the effects of protease inhibitors shortly after consumption, as a result of a long co-evolutionary history with their plant hosts that use these proteins as a defensive strategy against predation^9^,^10^,^11^. Whereas Cry toxins show strong toxicity towards relatively specific pests and allow for an effective insecticide-like effect in the field, protease inhibitors interfere with dietary protein digestion and lead, in the most potent cases, to amino acid shortage and a detrimental overexpression of digestive proteases causing growth delays and eventual death of the herbivore^12^. Most importantly, herbivorous insects have evolved a range of strategies to cope with dietary protease inhibitors, typically involving the secretion of complex midgut protease complements, the overexpression of inhibitor-sensitive proteases to outnumber the ingested inhibitors and the up-regulation of protease isoforms weakly sensitive to inhibition^8^. Nevertheless, and despite numerous unsuccessful attempts to use protease inhibitors in pest control, a number of promising cases have been reported recently^13^,^14^,^15^,^16^,^17^,^18^,^19^,^20^ that still remind the importance of digestive proteases in herbivorous pests and the possible relevance of these enzymes as effective targets for crop improvement^21^,^22^,^23^,^24^.

Protein engineering efforts have been made over the years to enhance the protective effects of plant protease inhibitors, notably involving fusion protein constructs to integrate complementary inhibitor domains into single polypeptides or the rational design of inhibitor variants with improved activity towards animal or plant protease models^25^. A practical challenge at present is to develop strategies for the selection of potent inhibitor candidates in such a way as to limit compensatory responses in the herbivore upon ingestion. Herbivorous arthropod genomes encode large families of digestive protease genes^26^,^27^,^28^ that allow the herbivores to produce protease isoforms with a wide range of affinity spectra towards dietary protein substrates and protease inhibitors^11^,^29^,^30^,^31^,^32^,^33^. Considering this, the most effective way to select useful inhibitors among a collection of available candidates may not be to test their inhibitory potency against one or a few model proteases, but to compare their effective binding range against the whole complement of possible protease targets in the pest midgut. In this study, we used a functional proteomics approach for the ‘capture’ and tandem mass spectrometry (MS/MS) analysis of protease inhibitor-susceptible proteases in crude biological extracts^33^ as a decision tool for the rational selection of a protease inhibitor useful to engineer resistance to the major coleopteran pest Colorado potato beetle (*Leptinotarsa decemlineata*) in potato, *Solanum tuberosum*. Unsuccessful attempts to implement resistance to this pest in potato varieties using recombinant protease inhibitors have been associated with the onset of multiple adaptive responses including increased leaf consumption to counterbalance the loss of digestive functions and overexpression of inhibitor-insensitive proteases to sustain basic protein digestion rates^34^,^35^,^36^. Here we show such compensatory responses to be compromised in larvae fed potato plants engineered to express a Cys protease inhibitor –or cystatin^37^– variant efficient in capturing midgut Cys proteases of this insect under our proteomics setup.

## Results and Discussion

### Tomato SlCYS8 variant P2V as a promising inhibitor for *L. decemlineata* control

**Figure 1:**
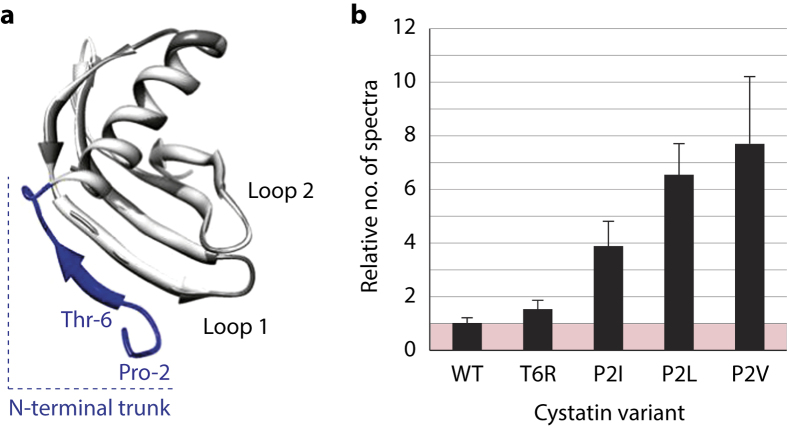
Spectral counts for intestain unique peptides captured with biotinylated versions of wild-type SlCYS8 and single functional variants P2I, P2L, P2V and T6R in midgut extracts of potato-fed *L. decemlineata* larvae. (**a**) Tertiary structure model for SlCYS8 (GenBank accession no. AF198390) showing the approximate positions of residues Pro-2 (P2) and Thr-6 (T6) targeted for mutagenesis in the N-terminal region, relative to the central (Loop 1) and C-terminal (Loop 2) inhibitory loops of the protein. The *in silico* model was generated with Modeller, v. 9.7^58^ using the NMR solution structure coordinates of oryzacystatin^59^ as a template (Protein Data Bank accession no. 1EQK). (**b**) Intestain unique peptides counted for the five SlCYS8 variants, as inferred from MS/MS datasets of refs 11 and 42. Data are expressed relative to total spectra counted for wild-type SlCYS8 (mean value adjusted to 1; *see* Supplementary Table S1 for details on unique peptide counts for each cystatin variant. Each bar is the mean of three independent (insect replicate) values ± se.

Tomato cystatin SlCYS8^38^ and single functional variants of this protein^39^ bearing an isoleucine (P2I), a leucine (P2L) or a valine (P2V) in place of the original proline at position 2, or an arginine (T6R) in place of the original threonine at position 6, were considered as possible candidates for potato transformation (Fig. 1a). *L. decemlineata* digestive Cys proteases in theory sensitive to cystatin inhibition, the so-called ‘intestains’^40^, are categorized into six functional families referred to, respectively, as intestains A (IntA), IntB, IntC, IntD, IntE and IntF^33^,^40^. Our proteomic approach consists of capturing cystatin-sensitive intestains in midgut extracts after binding biotinylated versions of the cystatins produced in *Escherichia coli* to an avidin-linked matrix for affinity enrichment^33^. After recovery, the captured intestains are detected as three bands of 25, 27 and 30 kDa on Coomassie blue-stained gels following SDS-PAGE, which include isoforms of the different functional families representing the complement of proteases bound by the cystatin variant. The three bands are excised, digested with trypsin, and the resulting peptides submitted to MS/MS for intestain identification and quantitation. The abundance of inhibitor variant-sensitive intestains in source extracts may be inferred by the counting of MS/MS unique peptide spectra, assuming a positive correlation between the number of captured peptides and the inhibitory range of the cystatin variant against specific intestain families or the whole range of intestains^33^,^41^.

We used this approach recently to address basic questions on the evolution and structure/function determinants of intestain–cystatin interactions in *L. decemlineata*^11^,^42^. An MS/MS peptides dataset generated during these studies was here reassessed to compare the ability of our SlCYS8 variant candidates to bind the insect intestains, assimilating the complement of captured proteases to the binding range of each variant. Thirty unique intestain peptides were detected overall following MS/MS, that could each be assigned to a single functional family (Supplementary Table S1). IntB-, IntC- and IntD-specific peptides were detected for all cystatin variants while no IntA-, IntE- or IntF-specific peptides were detected, presumably due to low numbers of intestains from these families in midgut extracts or to the reported insensitivity of some intestains to cystatin inhibition^40^. As inferred from total spectral counts, mean numbers of captured peptides differed from one variant to another, giving for instance mean numbers with P2V– and P2L–biotin six to seven times the peptide numbers obtained with the original SlCYS8 inhibitor (Fig. 1b). By comparison, T6R–biotin captured a smaller number of peptides, similar to the number captured with SlCYS8. These observations pointed overall to the usefulness of our functional proteomics approach to discriminate cystatin variants based on their effective binding range towards the whole complement of possible target Cys proteases in midgut extracts. They suggested, in practice, the potential of this approach to identify potent inhibitors, such as P2V in the present case, to implement insect resistance *in planta*.

### SlCYS8 variants P2V and T6R expressed in potato show differential effects on *L. decemlineata* larvae

**Figure 2:**
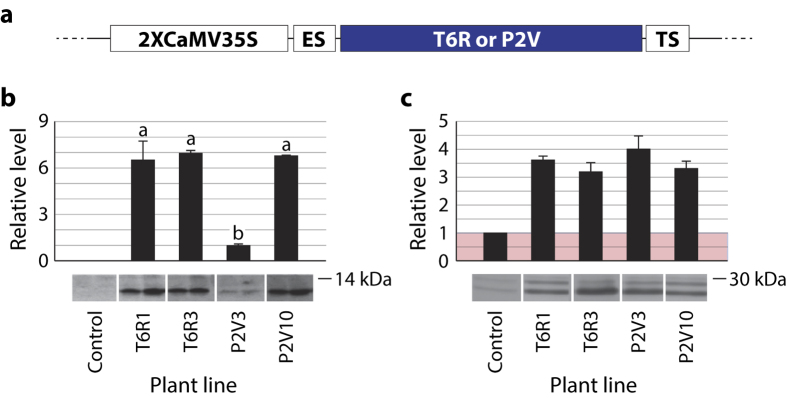
Recombinant cystatin content and relative amount of pathogen-inducible ß-glucanases (PR-2 proteins) in transgenic lines of potato expressing SlCYS8 variants T6R or P2V. (**a**) Gene construct elements for the cytosol-targeted expression of T6R and P2V in transgenic potato lines. Constructs included the basic coding sequence of either inhibitor^39^, a duplicated version of the Cauliflower mosaic virus (CaMV) 35 S promoter (2XCaMV35S) for constitutive expression in leaves, a tobacco etch virus enhancer sequence (ES) in upstream position of the cystatin coding sequence and a CaMV 35 S terminator sequence (TS) in downstream position. (**b**) Relative amounts of SlCYS8 variant in control (untransformed) and cystatin-expressing potato lines. Data are expressed relative to low-expressing line P2V3 (arbitrary value of 1.0). (**c**) Relative amounts of ß-glucanases in control and SlCYS8-expressing lines. Data are expressed relative to control line K (arbitrary value of 1.0). Numbers on the immunoblots indicate the position of commercial molecular mass markers (kDa). Each bar on panels b and c is the mean of three independent (plant replicate) values ± se. Bars with the same letter on panel b are not significantly different (post-anova Tukey’s HSD; α = 0.05).

P2V and T6R were used as candidate inhibitors for potato transformation (Fig. 2) to confirm a possible relationship between protease capture efficiency under our proteomics setup, insect resistance or susceptibility of transgenic plant lines expressing these inhibitors, and the relative ability of the target herbivore to mount an effective compensatory response upon leaf consumption. P2V was selected as an ‘effective inhibitor’ candidate based on the broad binding range of P2V–biotin towards intestains (*see* Fig. 1b) and previously reported data suggesting a strong inhibitory potency for this variant against *L. decemlineata* Cys proteases^39^. T6R was selected as a ‘weak inhibitor’ (negative control) candidate based on a narrow intestain binding range of T6R–biotin similar to the binding range of SlCYS8–biotin (Fig. 1b), despite previously reported inhibitory data with synthetic peptide substrates suggesting a greater potency of the single variant *in vitro*^39^. Potato lines engineered to express either inhibitors along with the antibiotic selection marker neomycin phosphotransferase II were regenerated on kanamycin-containing growth medium following genetic transformation with the appropriate gene constructs (Fig. 2a). *In vitro* clones produced from independent calli were acclimated in greenhouse and PCR-tested for the selection marker transgene in genomic DNA using appropriate DNA primers. A ~500-base-long ‘nptii’ amplicon was amplified from the DNA of all tested plants, confirming that all clones regenerated on kanamycin had been genetically transformed by the *Agrobacterium* transgene vector.

Immunodetections were performed with polyclonal IgG raised in rabbit against SlCYS8 to compare the relative amounts of recombinant P2V or T6R in transgenic leaves. As expected given the random insertion of transgenes in the genome of *Agrobacterium*-infected cells, immunoblot signals differed from one clone to another, from weak to moderate in leaves of low-expressing clones (such as P2V3) to strong or very strong in leaves of clones (such as T6R1, T6R3 or P2V10) expressing the cystatin at mean levels approximately seven times the mean level in line P2V3 (anova; *F*_(3,8)_ = 35.0, *P* = 0.001) (Fig. 2b). In agreement with studies reporting stress-related pleiotropic effects for recombinant cystatins in different plant species^7^ and the constitutive expression of naturally inducible pathogenesis-related (PR) proteins in potato lines expressing corn cystatin II^43^, ß-glucanases of the PR-2 family were up-regulated in leaves of cystatin-expressing clones compared to their steady-state levels in healthy leaves of parental line K (Fig. 2c). These observations confirmed the successful expression of T6R and P2V under an active form inducing pleiotropy in leaf tissue. They also pointed, given the accumulation of ß-glucanases at comparable rates among transgenic clones (*see* Fig. 2c), to the possible usefulness of T6R- and P2V-expressing lines as models to differenciate the expected protease inhibitory-mediated effects of P2V on *L. decemlineata* from eventual indirect effects via pleiotropic alterations of the endogenous defense system altering leaf tissue composition.

**Figure 3:**
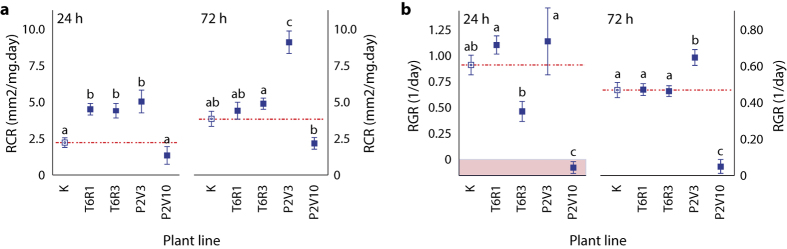
Relative leaf consumption and relative growth rates of *L. decemlineata* 4^th^ instars fed control line K or transgenic potato lines expressing T6R (T6R1 and T6R3) or P2V (lines P2V3 and P2V10). (**a**) Relative leaf consumption rates (RCR). (**b**) Relative growth rates (RGR). Data are expressed as mean values from six biological replicates ± se (n = 6 larvae), 24 h (1 d) or 72 h (3 d) after initiating the diet assay. Dashed lines indicate RCR and RGR values for control insects fed untransformed line K. Datapoints with the same letter(s) are not significantly different (post-anova Tukey’s HSD test; α = 0.05).

A feeding assay was conducted to compare the impacts of ectopically expressed P2V and T6R on growth and leaf consumption rates of *L. decemlineata* larvae (Fig. 3). Potato plants expressing either inhibitors were provided to 4^th^ instars over 72 h, a period of time sufficient for the larvae to adjust their protease complement to dietary protease inhibitors^36^. Short-term effects on foliage intake after 24 h were observed among larvae fed the different lines (anova; *F*_(4,25)_ = 78.0, *P* < 0.001) (Fig. 3a), giving relative consumption rates (RCR) for those larvae fed line T6R1, line T6R3 or line P2V3 about two times the RCR determined for larvae fed untransformed plants (post-anova Tukey’s HSD; *P* < 0.05). RCR values still differed after 72 h (anova; *F*_(4,25)_ = 219.5, *P* < 0.001) but increased RCR relative to control larvae at this time point were restricted to larvae fed the P2V-expressing line (post-anova Tukey’s HSD; *P* < 0.05). Similar trends were observed overall for larval growth after both 24 h (anova; *F*_(4,25)_ = 69.4, *P* < 0.001) and 72 h (anova; *F*_(4.25)_ = 409.1, *P* < 0.001) (Fig. 3b). As for consumption rates, relative growth rates (RGR) of larvae fed the two T6R-expressing lines were similar to the RGR of control larvae after 72 h (post-anova Tukey’s HSD; *P* > 0.05) but different from the RGR of larvae fed the P2V-expressing lines (*P* < 0.05). Diverging alterations of both the RCR and the RGR were observed for larvae fed the two P2V lines (post-anova Tukey’s HSD; *P* < 0.05) (Fig. 3a,b). As observed earlier with larvae given transgenic potato lines engineered to express the moderately efficient inhibitor oryzacystatin at high concentration^34^,^35^, line P2V3 (expressing P2V at low level) induced overcompensatory responses leading to increased larval growth and leaf consumption compared to control larvae. In sharp contrast, line P2V10 (expressing P2V at high concentration) had strong negative effects on the larvae, altering leaf consumption and compromising growth shortly after intake. These data indicated a strong, dose-dependent detrimental effect of P2V on *L. decemlineata* larvae and the practical usefulness of cystatin activity-based functional proteomics as a tool for the selection of protease inhibitors eventually useful in plant protection. They also suggested a limited impact of cystatin-mediated pleiotropic effects on the target insect, as inferred by the null effects of lines T6R1 and T6R3 on larval growth and food intake despite ß-glucanase inductions in leaves similar to those observed in the P2V lines.

### T6R- and P2V-expressing potato lines differentially alter digestive protease profiles in *L. decemlineata*

**Figure 4:**
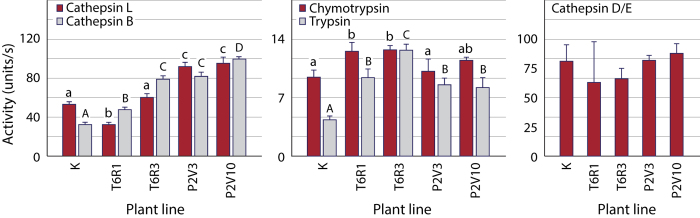
Cys (cathepsin L-like, cathepsin B-like), Ser (chymotrypsin-like, trypsin-like) and Asp (cathepsin D-like) protease activities in midgut extracts of *L. decemlineata* 4^th^ instars fed control line K or transgenic potato lines expressing T6R or P2V. Each bar is the mean of six independent (insect replicate) values ± se. Bars with the same letter are not significantly different (post-anova Tukey’s HSD; α = 0.05).

Protease assays were performed *in vitro* with family-specific fluorigenic peptide substrates to detect an eventual adjustment of the midgut protease complement in larvae fed cystatin-expressing lines, and to determine whether P2V and T6R had differential effects on Cys (e.g. intestain) and non-Cys protease activities after 72 h (Fig. 4). Cathepsin L-like Cys protease activity differed depending on the plant line provided (anova; *F*_(4,25)_ = 216.4, *P* < 0.001), with those larvae fed the P2V-expressing lines showing mean activity levels about two times the levels observed in larvae fed control or T6R-expressing lines (post-anova Tukey’s HSD test; *P* < 0.05). Cathepsin B-like Cys protease activity also differed depending on the plant (anova; *F*_(4,25)_ = 676.3, *P* < 0.001), again showing higher values for P2V-fed insects compared to the controls (post-anova Tukey’s HSD test; *P* < 0.05). As for Cys proteases, trypsin-like (anova; *F*_(4,25)_ = 37.2, *P* < 0.001) and chymotrypsin-like (anova; *F*_(4,25)_ = 7.06, *P* = 0.002) Ser protease activities in midgut extracts differed depending on the plant line, notably showing activity levels in T6R-fed larvae up to three times the activity observed in control larvae (post-anova Tukey’s HSD test; *P* < 0.05). By comparison, cathepsin D-like Asp protease activity did not vary with the plant line (anova; *F*_(4,25)_ = 1.10, *P* = 0.409), in contrast with a previously reported alteration of this activity in larvae fed potato lines expressing a tomato cathepsin D inhibitor^36^. These observations pointed overall to a broad compensatory adjustment of the midgut protease complement involving protease forms of at least two mechanistic classes in larvae fed the transgenic lines, including a cystatin variant-specific adjustment of cathepsin L-like (intestain) activity. The nature and the amplitude of protease adjustments were roughly similar in larvae fed the P2V3 or P2V10 lines despite the diverging effects of these two lines on growth or leaf consumption rates (*see* Fig. 3). This observation, although surprising at first sight, may simply reflect the priority given to digestive proteases in herbivorous insects and the ability of these organisms to readily adjust their protease complement upon ingestion of antidigestive compounds to sustain basic digestive functions.

**Figure 5:**
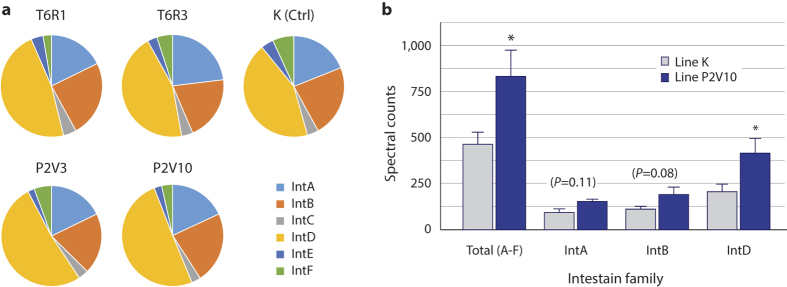
MS/MS intestain peptide spectral counts in midgut extracts of *L. decemlineata* larvae fed control line K or transgenic potato lines expressing T6R or P2V. (**a**) Relative distribution of intestain family-specific peptides among intestain functional families A to F (*see* Supplementary Table S2 for details on intestain peptides). (**b**) Total intestain, IntA-specific, IntB-specific and IntD-specific spectral counts for larvae fed control line K or cystatin-expressing line P2V10. Data are expressed as total spectral counts overall (Total) or total spectral counts for each intestain family (*see* Table 1 and Supplementary Table S2 for details on unique peptide counts). Each bar is the mean of three independent (insect replicate) values ± se. Asterisks indicate statistically different values between diet treatments (post-anova Tukey’s HSD; *P*  <  0.05).

A shotgun proteomics procedure was used to compare intestain profiles in P2V- and T6R-line fed larvae and to determine whether altered Cys protease activities after transgenic line ingestion were associated with a general effect on the intestains or, instead, with the targeted induction of specific intestain families (Fig. 5 and Table 1). The procedure was similar to the procedure described above for cystatin-captured intestains, except that no capture step was carried out prior to migration and recovery of the protein bands in Coomassie blue-stained gels. One hundred and thirty-two unique intestain peptides were detected following MS/MS, that could each be assigned to a single intestain family (Supplementary Table S2). Intestain members of the six functional families were found at similar relative rates in control and cystatin-fed larvae after 72 h, with IntA, IntB and IntD isoforms being by far the most abundant regardless of the plant line provided (Fig. 5a). In accordance with the above-described protease assays showing plant line-dependent alterations of cathepsin B- and cathepsin L-like protease activities, total numbers of intestain peptide spectral counts differed from line to another (anova; *F*_(4,10)_ = 4.87, *P* = 0.023), with midgut extracts of P2V10 line-fed larvae containing the largest number of peptides overall (post-anova Tukey’s HSD; *P* < 0.05) (Table 1). Peptide spectral counts for all intestain families, except the IntE family (anova; *F*_(4,10)_ = 2.02, *P* = 0.168), were influenced to some extent by the plant line, with levels in midgut extracts of larvae fed the T6R1 line often lower than those of P2V line-fed larvae (post-anova Tukey’s HSD; *P* < 0.05) (Table 1). More specifically, total spectral counts and spectral counts specific to the abundant IntA, IntB and IntD isoforms in larvae fed the P2V10 line were about twice the spectral counts observed in line K-fed control insects (Fig. 5b). These data suggested a general, intestain family-independent up-regulation of the intestain complement after ingestion of the P2V-expressing lines. They also supported the hypothesis of a link between protease inhibitor (e.g. cystatin) detrimental effects and digestive protease (e.g. intestain) overexpression upon inhibitor intake, as proposed earlier for lepidopteran pests given Ser protease inhibitors at high concentration^12^.

## Conclusion

Our main goal in this study was to assess the potential of a cystatin activity-based functional proteomics approach to identify cystatin variants eventually useful in plant protection. Current procedures to compare the potency of protease inhibitors against herbivore digestive proteases generally rely on *in vitro* protease inhibitory assays with synthetic peptide substrates to determine dissociation constants (*K*_d_) towards one or a few model proteases, or to estimate threshold inhibitory concentration values (e.g. *IC*_50_ values) towards specific protease functional families in midgut extracts^44^. Such measurements give useful information about the relative potency of different inhibitors against specific proteases or protease subsets, but they say little about the actual inhibitory range of these proteins towards the whole complement of possible protease targets in the herbivore pest. As importantly, diagnostic peptide substrates assumed to be specific to a given protease family may sometimes be resistant to protease isoforms of this family or, on the contrary, be susceptible to protease isoforms of alternative families. By comparison, functional proteomics approaches such as the activity-based procedure adopted herein to capture inhibitor-sensitive proteases provide a more realistic picture of protease–inhibitor interactions that take place in source extracts, with no masking or confounding effects causing an over- or underestimation of protease binding ranges. An obvious case of discrepancy for a same set of samples characterized using the two experimental approaches was here provided with T6R and P2V. These two cystatin variants were suggested previously to present a similar inhibitory range towards midgut Cys proteases of *L. decemlineata* larvae, based on *in vitro* assay data produced with synthetic peptide substrates^39^. In sharp contrast, the P2V–biotin fusion captured almost five times more intestain peptides than its T6R counterpart (*see* Fig. 1), pointing in fact to significantly different protease binding ranges for the two cystatins and a likely overestimation of protease targets for T6R as assessed with commonly used *in vitro* assays.

The high potency of P2V in capturing intestains in *L. decemlineata* midgut extracts was associated with significant short-term effects of transgenic potato lines expressing this inhibitor on larval growth and leaf consumption rates. Studies assessing the long-term detrimental effects of P2V-expressing potato lines on *L. decemlineata* throughout its life cycle^34^,^35^ and studies with alternative inhibitors of Cys proteases (e.g. ref. 45), host plants and herbivorous pests will be welcome in coming years to further confirm the potential of functional proteomics as a predictive tool for the selection of protease inhibitors useful in crop protection. Studies will also be welcome to explore the potential of inhibitor activity-based procedures for the selection of effective Ser protease inhibitors. Many papers have described the diversity of midgut chymotrypsin- and trypsin-like enzymes in lepidopteran insects^26^, and the hurdles still to be overcome to confirm the general usefulness of Ser protease inhibitors in plant protection^8^.

## Methods

### Transgenic plant lines

Transgenic potato lines (*Solanum tuberosum*, cv. Kennebec) expressing T6R or P2V were produced by *Agrobacterium tumefaciens*-mediated transformation of ‘line K’ axenic plantlets^46^ and selected *in vitro* using the neomycin phosphotransferase II selection marker for kanamycin resistance, as described earlier^47^. Gene constructs for transformation consisted of either cystatin-encoding sequences^39^ introduced between the *NcoI* and *BsrGI* cloning sites of a modified pUC19 vector (Fermentas Life Science) harbouring a duplicated version of the Cauliflower mosaic virus (CaMV) 35 S promoter, a tobacco etch virus enhancer sequence and the CaMV 35 S terminator sequence. The resulting constructs were transferred into the pCambia 2300 vector (CAMBIA) for plant genetic transformation (*see* Fig. 2a). Regenerated plantlets were acclimated for 14 days in a growth chamber under a 24°/21 °C day/night temperature cycle, a 12:12 h light to dark photoperiod, a light intensity of 175 μmol.m^−2^.s^−1^ and a relative humidity of 60%, before their transfer in greenhouse for multiplication and further analysis. Integration of the *nptii* selection transgene in kanamycin-resistant plants was confirmed by PCR using DNA extracted from the fifth leaf of 30-cm tall potato plants [down from the apex] according to Edwards *et al*.^48^. The following primers were used for amplification: 5′–ACTGA AGCGG GAAGG GACTG GCTGC TATTG, and 3′–GATAC CGTAA AGCAC GAGGA AGCGG TCAG. The transgene amplicon was visualized by ethidium bromide staining after resolving the PCR products (~500 bases) in 1% (w/v) agarose gels.

### Cystatins and ß-glucanases in leaves

T6R, P2V and ß-glucanases (PR-2 proteins) were immunodetected in total soluble protein extracts prepared from the fifth leaf of 30 cm-tall plants, down from the apex. Leaf soluble proteins were extracted in mild conditions as described earlier^49^ and resolved by 12% (w/v) SDS-PAGE using the Bio-Rad Mini Protean III Electrophoresis Unit™ (Bio-Rad). The proteins were electrotransferred onto Hybond ECL nitrocellulose sheets (GE Healthcare) using the Bio-Rad Mini-transfer Unit™, according to the supplier’s instructions. T6R and P2V were immunodetected with commissioned polyclonal IgG raised in rabbits against SlCYS8 (AgriSera), ß-glucanases with commercial polyclonal IgG raised in rabbits against tobacco PR-2 proteins (AgriSera), and primary IgG with goat anti-rabbit IgG conjugated to alkaline phosphatase. Protein–antibody complexes were visualized using the alkaline phosphatase substrate 5-bromo-4-chloro-3-indolyl phosphate and nitro blue tetrazolium for colour development (Life Technologies). Densitometric analysis of the protein signals on nitrocellulose sheets was performed with the Phoretix 2D Expression software, v. 2005 (NonLinear USA) after scanning the immunoblots with an Amersham Image Scanner digitalizer (GE Healthcare). All immunodetections involved three independent (biological) replicates to allow for statistical analyses.

### Insect feeding assay

Synchronized 4^th^ instars were used for the insect feeding assay, derived from a laboratory colony collected on field-grown potato plants at Laval University experimental station near Québec City QC, Canada. The experimental setup was kept at 20 °C and 65% relative humidity in a PGw36 growth chamber (Conviron), under a 16 h daily photoperiod. Three plants of each line were distributed randomly in the growth chamber and two larvae were assigned to each plant, i.e. one on the 5^th^ leaf and one on the 6^th^ leaf. Individual larvae (n = 6) and leaves (n = 6) were monitored after 24 h (1 d) and 72 h (3 d) to estimate larval relative growth rates (RGR) and relative consumption rates ×(RCR)^34^, based on the following equations:


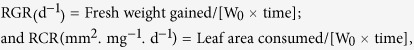


where W_0_ is the mean fresh weight of each group of six larvae at time 0 (mg) and time the number of days (d) after starting the assay. Individual larvae were weighed using an MT5 microbalance (Mettler Toledo). Foliage consumption corresponded to total leaf surface eaten (mm^2^) as estimated using a 1-cm^2^ paper disc guide^36^. Larvae were collected and dissected at the end of the 3-d assay, and their midgut frozen at –80 °C until use for enzymology and proteomic analyses.

### Insect midgut proteins

Midgut proteins for protease assays and proteomic profiling were extracted in 100 mM citrate phosphate extraction buffer, pH 6.0, containing 10% (v/v) ethylene glycol. Snap-frozen insect samples were first ground to a fine powder in liquid nitrogen and kept on ice for 10 min in three volumes of extraction buffer. Protein mixtures were clarified by centrifugation at 15,000 *g* for 10 min at 4 °C, and the supernatant used as source material for further analysis. Protein content in the extracts was adjusted to 2 μg/μl by the addition of extraction buffer, after assaying soluble proteins according to Bradford^50^ with bovine serum albumin as a protein standard.

### Protease assays

Protease activities were determined by the monitoring of fluorigenic peptide hydrolysis progress curves as described earlier^39^. Cathepsin B-like activity was measured at pH 6.5 in 100 mM sodium phosphate containing 10 mM l-cysteine, with *Z*–Arg-Arg–methylcoumarin (MCA) (Peptides International) as a substrate. Cathepsin L-like activity was measured in the same buffer with *Z*–Phe-Arg–MCA (Peptides International) as a substrate. Cathepsin D-like activity was measured at pH 3.0 in 50 mM citrate phosphate with the substrate MOCAc-Gly–Lys–Pro–Ile–Leu–Phe–Phe–Arg–Leu–Lys(Dnp)-d-Arg-NH_2_ (Peptides International). Trypsin-like activity was measured in 100 mM sodium phosphate buffer, pH 7.5, with the substrate *Z*–Arg–MCA (Peptides International). Chymotrypsin-like activity was measured in 100 mM sodium phosphate, pH 7.5, with the substrate Glt–Ala–Ala–Phe–MCA (TPCK-leupeptine) (Peptides International). Substrate fluorescence upon cleavage was detected using a Fluostar Galaxy reader (BMG Labtech), with excitation and emission filters of 360 and 450 nm, respectively, for the MCA substrates; or of 340 and 400 nm, respectively, for the MOCAc substrate.

### Mass spectrometry

Intestains were identified by liquid chromatography (LC)-MS/MS analysis of intestain bands recovered from Coomassie blue-stained polyacrylamide slab gels. Thirty μg of midgut protein was resolved by 12% (w/v) SDS-PAGE and gel slices encompassing protein bands in the ~25–32-kDa range were carefully excised^33^. The gel slices were destained, reduced in 10 mM dithiothreitol, alkylated in 55 mM iodoacetamide and hydrolyzed for 1 h at 58 °C with 125 nM TrypsinGold (Promega) using a MassPrep Workstation robot (Waters-Micromass)^51^. Peptides in the gel matrix were extracted in 2% (v/v) acetonitrile (Acn):1% (v/v) formic acid and then washed several times in 50% (v/v) Acn:1% (v/v) formic acid. The extracts were pooled, vacuum centrifuged and resuspended in 7 μl of 0.1% (v/v) formic acid, from which 2 μl was taken for LC-MS/MS analysis. The peptides were resolved by reversed-phase nanoscale capillary LC and then submitted to electrospray MS. A Thermo Surveyor MS pump was used, connected to an LTQ linear ion trap mass spectrometer equipped with a nanoelectrospray ion source (ThermoFisher). Peptide separation was performed on a PicoFrit column (NewObjective) packed with Jupiter 5 μ C18 300 A bulk packing (Phenomenex), at 200 nl/min [obtained by flow splitting] over 30 min along a linear gradient going from 2 to 50% (v/v) Acn:0.1% (v/v) formic acid. MS/MS data were acquired under the data-dependent acquisition mode using the Xcalibur software, v. 2.0 (Thermo Scientific). The seven most intense ions in the 400–2,000 *m/z* range were selected for collisional induced fragmentation, with the dynamic exclusion function enabled, an exclusion duration of 30 s and relative collisional fragmentation energy set at 35.

### Protease identification

MS peaklists generated from the Xcalibur raw data using Mascot Distiller v. 2.3 (Matrix Science) were analyzed with Mascot 2.3.02 (Matrix Science)^52^ set up to search the Uniref100 database (http://uniprot.org/uniref/)^53^. Search parameters for protein matching were as follows: a fragment ion mass tolerance of 0.5 Da, a parent ion tolerance of 2.0 Da, iodoacetamide derivatives Cys residues as fixed modification, oxidized Met residues as variable modification, and a maximum of two missed trypsin cleavages allowed. MS/MS based peptide and protein identifications were validated with Scaffold, v. 3.6.1 (Proteome Software). Peptide identifications were accepted if they could be established at greater than 95% probability as specified by the Peptide Prophet algorithm^54^. Protein identifications were accepted if they included at least two identified unique peptides and could be established at greater than 95% probability using the Protein Prophet algorithm^55^. Proteins that contained similar peptides and could not be differentiated based on MS/MS spectra were grouped to satisfy the principle of parsimony.

### Spectral count analyses

Quantitative analysis of MS spectra was done using spectral count sampling statistics^56^ on those counts corresponding to peptides that were specific to an intestain functional family^33^. Spectra obtained from the individual bands were combined for each repetition, and only those belonging to peptides specific to an intestain family were included in the quantitative analysis. The differential rates of identified intestains were discriminated statistically with a significance threshold of 5%, taking into account spectral count mean values greater than 5 for at least one treatment^57^.

## Additional Information

**How to cite this article**: Rasoolizadeh, A. *et al*. Functional proteomics-aided selection of protease inhibitors for herbivore insect control. *Sci. Rep.* **6**, 38827; doi: 10.1038/srep38827 (2016).

**Publisher's note:** Springer Nature remains neutral with regard to jurisdictional claims in published maps and institutional affiliations.

## Supplementary Material

Supplementary Information

## Figures and Tables

**Table 1:** MS/MS intestain peptide spectral counts in midgut extracts of *L. decemlineata* larvae fed control line K or transgenic potato lines expressing T6R (lines T6R1 and T6R3) or P2V (lines P2V3 and P2V10).

Peptides	No. spectral counts				
	Line K	Line T6R1	Line T6R3	Line P2V3	Line P2V10
IntA-specific	89 ± 16 ab	60 ± 11 a	97 ± 20 ab	99 ± 15 ab	150 ± 15 b
IntB-specific	108 ± 16 ab	82 ± 19 a	86 ± 11 a	109 ± 13 ab	190 ± 43 b
IntC-specific	17 ± 1.2 a	14 ± 0.6 a	15 ± 1.7 a	18 ± 0.2 ab	24 ± 0.9 b
IntD-specific	204 ± 43 a	159 ± 27 a	188 ± 29 a	287 ± 49 ab	417 ± 81 b
IntE-specific	19 ± 3.5	13 ± 0.7	13 ± 4.7	12 ± 1.0	20 ± 0.7
IntF-specific	32 ± 5.2 a	9 ± 2.9 b	21 ± 2.1 ab	31 ± 2.4 a	29 ± 6.4 a
Total	469 ± 67 ab	337 ± 59 a	420 ± 60 ab	556 ± 76 b	830 ± 140 c

Data are expressed as total spectral counts for each intestain family (IntA to IntF) or total spectral counts overall (Total) (see Supplementary Table S2 for details on unique peptide counts). Each value is the mean of three independent (insect replicate) counts ± se. On each line, data with the same letter(s) are not significantly different (post-anova Tukey’s HSD; α = 0.05).
