# Supplementary material for: Functional proteomics-aided selection of protease inhibitors for herbivore insect control
Source: Sci Rep. 2016 Dec 13;6:38827. doi: 10.1038/srep38827 (PMC5153846; doi:10.1038/srep38827)
Supplement: Supplementary Information [file srep38827-s1.pdf]

Supplementary Information | Tables S1 and S2

Functional proteomics-aided selection of protease inhibitors for herbivore insect control

Asieh Rasoolizadeh[1], Aurélie Munger[1], Marie-Claire Goulet[1], Frank Sainsbury[1,*], Conrad Cloutier[2] & Dominique Michaud[1]

[1] Département de phytologie, Université Laval, Québec City, QC, Canada.

[2] Département de biologie, Université Laval, Québec City QC, Canada.

* Present address: Australian Institute for Bioengineering and Nanotechnology, The University of Queensland, St. Lucia QLD, Australia.

Supplementary Table S1 Numbers of intestain unique peptides detected following MS/MS analysis of intestains captured with SlCYS8 variants in a midgut extract of *L. decemlineata* larvae raised on potato, cv. Kennebec [a]

No. peptides [b]	SlCYS8 variant					
	wt	T6R	P2I	P2L	P2V	Overall
No. distinct unique peptides [c]						
. IntB-specific	3	6	13	13	13	14
. IntC-specific	2	2	2	3	3	3
. IntD-specific	1	2	5	8	12	13
. Total	6	10	20	24	28	30
No. peptide spectral counts [c]						
. IntB-specific	15	20	52	61	108	256
. IntC-specific	2	6	6	7	7	28
. IntD-specific	1	3	12	51	23	90
. Total	18	29	70	119	138	374
. Relative number ('wt' basis) [d]	1.0	1.6	3.9	6.6	7.7	

[a] Data inferred from MS/MS datasets of refs. 1 and 2, below.

[b] No IntA-, IntE- or IntF-specific peptides detected under our conditions.

[c] Mean total numbers for three biological (insect) replicates.

[d] See Fig. 1b for a graphical representation of inter-replicate variation.

1 Vorster, J. *et al.* Positive selection of digestive Cys proteases in herbivorous Coleoptera. *Insect Biochem. Mol. Biol.* **65,** 10–19 (2015).

2 Rasoolizadeh, A., Goulet, M.C., Sainsbury, F., Cloutier, C. & Michaud, D. Single substitutions to closely related amino acids contribute to the functional diversification of an insect-inducible, positively selected plant cystatin. *FEBS J.* **283,** 1623–1635 (2016).

Supplementary Table S2 Unique intestain peptides detected by MS/MS in midgut extracts of *Leptinotarsa decemlineata* 4th instars fed control line K or cystatin-expressing transgenic potato lines T6R1, T6R3, P2V3 or P2V10 [a]

Family	Unique peptides	Spectral counts [b]					Accession numbers
		K	T6R1	T6R3	P2V3	P2V10	
**A**	DANNLCDIASMCSYPILL	0	3	1	3	3	Q81889; Q6QRP8
	DLELPEQIDWTEK	0	0	0	0	2	Q8I887; Q6QRP8
	DLELPEQIDWTEKGAVLPVK	0	0	0	0	1	Q8I887
	GAVLPVKNQGNCR	18	13	19	21	28	Q8I887
	KLLADEDELK	4	0	0	0	0	Q6QRP8; Q8I889
	KLLADEDELKK	16	16	20	20	38	Q6QRP8; Q8I889
	KTIVQIKGYK	0	0	2	0	4	Q6QRP8; Q8I889
	LLADEDELK	6	0	2	0	4	Q6QRP8; Q8I889
	LLADEDELKK	28	20	24	30	30	Q6QRP8; Q8I889
	LPEQIDWTEK	6	4	6	20	20	Q8I887; Q6QRP8
	LPEQIDWTEKGAVLPVK	0	2	0	1	0	Q8I887
	LPEQIDWTEKGAVLPVKNQGNCR	6	7	7	8	8	Q8I887
	LPVKNQGNCR	0	0	0	1	0	Q8I887
	NSWGTTWGEDGYFR	16	12	18	12	24	Q6QRP8; Q8I889
	NSWGTTWGEDGYFRIER	46	24	50	42	48	Q6QRP8; Q8I889
	SCWAFSTTGSLEGQNAIHNK	56	22	66	42	116	Q8I887; Q6QRP8
	SCWAFSTTGSLEGQNAIHNKVK	20	14	18	18	44	Q8I887; Q6QRP8
	TIVQIKGYK	2	4	6	8	12	Q6QRP8; Q8I889
	TIVQIKGYKK	8	8	10	12	12	Q6QRP8; Q8I889
	VKNSWGTTWGEDGYFR	16	8	18	18	24	Q6QRP8; Q8I889
	VKNSWGTTWGEDGYFRIER	44	22	50	34	64	Q6QRP8; Q8I889

**B**	AVGTVGPVSVAIDADPIQLYSGGILDGLF CTHNLNHGVLAVGYGEEDHLFGK	2	0	0	0	0	Q6QRP6; Q8I884
	DANNLCGIADK	46	34	34	38	44	Q6QRP6; Q8I884
	DANNLCGIADKA	2	0	0	0	0	Q6QRP6; Q8I884
	DANNLCGIADKASYPIL	18	14	20	16	52	Q6QRP6; Q8I884
	DTPCQYDAKK	4	2	6	4	2	Q6QRP6; Q8I884
	DWGEQGYFR	22	12	14	14	18	Q6QRP6; Q8I884
	EGLEVPDSIDWTQKGAVLDVK	0	0	0	1	0	Q6QRP6
	EVPDSIDWTQK	10	7	4	8	8	Q6QRP6; Q8I883
	EVPDSIDWTQKGAVLDVK	1	0	0	0	0	Q6QRP6
	GAVLDVKYQGGCGSCWAFSATGA LEGQNAIVNNVK	3	1	0	0	6	Q6QRP6
	GAVLGVKYQGGCGSCWAFSATGA LEGQNAIVNNVK	1	0	0	0	1	Q8I883
	GIDTPCQYDAK	17	10	13	19	21	Q6QRP6
	GIDTPCQYDAKK	13	4	10	13	34	Q6QRP6
	GIDTPCQYDAKKTVLK	3	4	5	7	8	Q6QRP6
	GIEADSSYPYK	28	20	24	28	26	Q6QRP6; Q8I884
	GIEADSSYPYKGIDTPCQYDAK	6	4	3	6	7	Q6QRP6
	GIEADSSYPYKGIDTPCQYDAKK	6	6	6	7	14	Q6QRP6
	GYKNVSISEEELKK	0	0	0	0	1	Q8I884
	IKRDANNLCGIADK	10	8	8	8	12	Q6QRP6; Q8I884
	IPLSEQQLLDCSKPYGNDDCEHGGL MSFAFDYVLDK	6	0	6	8	12	Q6QRP6; Q8I884
	IPLSEQQLLDCSKPYGNDDCEHGGL MSFAFDYVLDKGIEADSSYPYK	10	6	4	6	12	Q6QRP6; Q8I884
	LEVPDSIDWTQK	0	0	0	0	2	Q6QRP6; Q8I883
	NSWGKDWGEQGYFR	26	24	24	32	84	Q6QRP6; Q8I884
	NVSISEEELK	2	0	0	0	4	Q6QRP6; Q8I884

**B**	NVSISEEELKK	6	0	4	4	26	Q6QRP6; Q8I884
	RDANNLCGIADK	8	6	2	10	12	Q6QRP6; Q8I884
	RDANNLCGIADKASYPIL	8	10	10	12	14	Q6QRP6; Q8I884
	VKNSWGKDWGEQGYFR	14	20	14	8	80	Q6QRP6; Q8I884
	YQGGCGSCWAFSATGALEGQNAIVN NVK	16	8	12	12	22	Q6QRP6; Q8I883
**C**	AAVQIKSYK	0	0	0	0	2	Q6QRP5
	AIKKNDEIDLQK	3	5	4	7	5	Q6QRP5
	AVALEGPVSVAIEVTIAFQLYAR	21	0	1	1	3	Q6QRP5
	DGDLPAEVDWTK	1	1	1	0	0	Q6QRP5
	DLPAEVDWTK	0	0	0	1	0	Q6QRP5
	FDGDLPAEVDWTK	0	1	1	0	1	Q6QRP5
	FNNSKAAVQIK	0	0	0	0	1	Q6QRP5
	GILNDPQCK	12	10	10	11	10	Q6QRP5
	GILNDPQCKNTEGDLTHAVLVTGYG SQDGKDYWIVK	0	1	1	0	2	Q6QRP5
	LISLSEQQLVDCVK	6	6	6	7	12	Q6QRP5
	NDEIDLQK	2	0	2	4	1	Q6QRP5
	NSWGAEYGMDGYLR	8	6	7	6	7	Q6QRP5
	NTEGDLTHAVLVTGYGSQDGKDYWIVK	6	4	4	4	14	Q6QRP5
	SQGSCGSCWAFSTTGSVESHNFIK	5	0	1	3	4	Q6QRP5
	TGKLISLSEQQLVDCVK	0	0	0	0	1	Q6QRP5
**D**	ASQWSGETKFWR	36	39	45	48	126	A2I7P3; A2I7P2; A2I7P9
	ASQWSGETKFWRVK	0	0	0	0	2	A2I7P3; A2I7P2
	AVGTIDPISIAMNSDPLQLYYSGIISGK	1	0	0	0	0	A2I7Q3
	AVGTIGPISIAMNSDPLQLYYSGIISGK	8	5	5	10	6	A2I7P6
	DANNLCGIADDPTYPVL	12	16	20	16	52	A2I7P6; A2I7P3; A2I7P2; A2I7P9

**D**	DLEVPDSIDWTEK	0	0	0	0	3	A2I7Q3; A2I7P3; A2I7P2
	DQNPCGSCWAFSATGALEGQNAIL NNVK	4	0	2	0	0	A2I7Q3; A2I7P3
	DYGIQSEKSYPYIR	12	12	18	18	45	A2I7Q3; A2I7P3; A2I7P2
	DYGIQSEKSYPYIRK	27	15	15	27	36	A2I7Q3; A2I7P3; A2I7P2
	EGGDMSAAFEYVR	24	12	12	12	12	A2I7Q3; A2I7P3
	EGGDMSAAFEYVRDYGIQSEK	16	14	10	12	16	A2I7Q3; A2I7P3
	EGGDMSAAFEYVRDYGIQSEKSYPYIR	4	0	4	0	8	A2I7Q3; A2I7P3
	EVPDSIDWTEK	15	12	6	15	9	A2I7Q3; A2I7P3; A2I7P2
	FPEDLEVPDSIDWTEK	0	0	0	0	3	A2I7Q3; A2I7P3; A2I7P2
	GAVLEVKDQNPCGSCWAFSATGALE GQNAILNNVK	14	8	12	14	18	A2I7Q3; A2I7P3
	GAVLEVKDQNPCGSCWAFSATGALK GQNAILNNVK	0	2	0	0	2	A2I7P2
	GCSHDLDHGVLVVGYGK	52	32	28	60	100	A2I7P6; A2I7P3; A2I7P2; A2I7P9
	GCSHDLDHGVLVVGYGKASQWSGET KFWR	0	0	0	0	3	A2I7P3; A2I7P2; A2I7P9
	GYKNVTTSEEGLRK	6	0	6	9	9	A2I7Q3; A2I7P3; A2I7P2
	IKRDANNLCGIADDPTYPVL	8	16	20	24	28	A2I7P6; A2I7P3; A2I7P2; A2I7P9
	ISLSEQQLLDCSAAYGNGNCK	24	18	18	24	51	A2I7Q3; A2I7P3; A2I7P2
	ISLSEQQLLDCSAAYGNGNCKEGGD MSAAFEYVRDYGIQSEK	2	0	0	0	4	A2I7Q3; A2I7P3
	IWGENGYFR	124	80	120	20 8	256	A2I7P6; A2I7P3; A2I7P2; A2I7P9
	KAVGTIGPISIAMNSDPLQLYYSGII SGK	8	1	4	6	4	A2I7P6
	KAVGTIGPISIAMNSDPLQLYYSGTI SGK	1	0	0	1	2	A2I7P2
	KGYKNVTTSEEGLR	0	0	0	3	0	A2I7Q3; A2I7P3; A2I7P2

**D**	KQTECQYDASKTILK	33	24	30	39	81	A2I7Q3; A2I7P3; A2I7P2
	LEVPDSIDWTEK	0	3	0	0	0	A2I7Q3; A2I7P3; A2I7P2
	NSWGKIWGENGYFR	34	34	33	35	86	A2I7P6; A2I7P3; A2I7P2; A2I7P9
	NVTTSEEGLR	0	0	0	0	3	A2I7Q3; A2I7P3; A2I7P2
	NVTTSEEGLRK	9	0	6	12	9	A2I7Q3; A2I7P3; A2I7P2
	QTECQYDASK	3	0	0	6	6	A2I7Q3; A2I7P3; A2I7P2
	QTECQYDASKTILK	21	27	30	39	54	A2I7Q3; A2I7P3; A2I7P2
	QTECQYDASKTILKIK	0	0	0	0	3	A2I7Q3; A2I7P3; A2I7P2
	RDANNLCGIADDPTYPVL	16	0	20	24	28	A2I7P6; A2I7P3; A2I7P2; A2I7P9
	VKNSWGKIWGENGYFR	18	9	15	18	36	A2I7P6; A2I7P3; A2I7P2
	VPDSIDWTEKGAVLEVK	3	3	3	3	3	A2I7Q3; A2I7P3; A2I7P2
	VQNSWGKIWGENGYFR	7	7	6	8	29	A2I7P9
**E**	AKGIEAGSSYPYQGR	6	6	4	6	11	A2I7N8
	AVGTIGPISVAVSSEHLR	11	9	11	7	18	A2I7N8
	CIKDLDHAVLAVGYGSENGR	6	1	0	0	3	A2I7N8
	CIKDLDHAVLAVGYGSENGRK	1	0	0	0	0	A2I7N8
	DAGNLCGVASMASYPLL	0	0	2	0	2	A2I7N8
	GIEAGSSYPYQGR	10	7	7	10	15	A2I7N8
	KAVGTIGPISVAVSSEHLR	6	1	3	0	0	A2I7N8
	LYGGGVITTR	5	4	4	4	3	A2I7N8
	TWGDHGYFK	4	3	4	2	2	A2I7N8
**F**	AVGTIGPISIAMNADPLQLYFGGILSGR	3	0	1	0	0	A2I7Q1
	DANNLCGIADDPTYPVL	3	4	5	4	13	A2I7P5
	DLEVPDSIDWTEK	0	0	0	0	1	A2I7Q2

**F**	DYGIEAEESYPYK	6	0	5	6	2	A2I7Q1; A2I7Q2
	EVPDSIDWTEK	5	0	2	5	2	A2I7Q1
	FPEDLEVPDSIDWTEK	0	0	0	0	1	A2I7Q2
	GCSDELDHAVLAVGYGEVSQSSAK	6	4	4	6	7	A2I7P7
	GCTDELDHGVLAVGYGEVSQSSGNTK	4	0	1	0	0	A2I7P5
	GCTDELDHGVLAVGYGEVSQSSGNT KFWK	7	0	1	5	2	A2I7P5
	IKRDANNLCGIADDPTYPVL	2	4	5	6	7	A2I7P5
	NSWGDYWGEDGYFR	6	4	4	6	9	A2I7P7
	NSWGDYWGEKGYFR	6	0	4	4	2	A2I7P5
	RDANNLCGIADDPTYPVL	4	0	5	6	7	A2I7P5
	SIETVCQYDASK	9	0	6	7	2	A2I7Q1; A2I7Q2
	SIETVCQYDASKTILK	6	0	2	5	3	A2I7Q1; A2I7Q2
	VKNSWGDYWGEDGYFR	6	4	4	6	16	A2I7P7
	VKNSWGDYWGEK	5	0	0	1	0	A2I7P5
	VKNSWGDYWGEKGYFR	8	1	6	7	5	A2I7P5

[a] Mean spectral counts for three biological (insect) replicates.
